# Cardiac dimensions and hemodynamics in healthy juvenile Landrace swine

**DOI:** 10.1186/s12947-023-00321-9

**Published:** 2024-01-16

**Authors:** Michelle Costa Galbas, Hendrik Cornelius Straky, Florian Meissner, Johanna Reuter, Marius Schimmel, Sebastian Grundmann, Martin Czerny, Wolfgang Bothe

**Affiliations:** 1 Department of Cardiovascular Surgery, Heart Center Freiburg—Bad Krozingen, Faculty of Medicine, University of Freiburg, Hugstetter Strasse 55, 79106 Freiburg, Germany; 2 Department of Cardiology and Angiology, Heart Center Freiburg, Faculty of Medicine, University of Freiburg, Freiburg, Germany

**Keywords:** Epicardial echocardiography, Experimental animal models, Hemodynamics, Swine

## Abstract

**Background:**

Swine are frequently used as animal model for cardiovascular research, especially in terms of representativity of human anatomy and physiology. Reference values for the most common species used in research are important for planning and execution of animal testing. Transesophageal echocardiography is the gold standard for intraoperative imaging, but can be technically challenging in swine. Its predecessor, epicardial echocardiography (EE), is a simple and fast intraoperative imaging technique, which allows comprehensive and goal-directed assessment. However, there are few echocardiographic studies describing echocardiographic parameters in juvenile swine, none of them using EE. Therefore, in this study, we provide a comprehensive dataset on multiple geometric and functional echocardiographic parameters, as well as basic hemodynamic parameters in swine using EE.

**Methods:**

The data collection was performed during animal testing in ten female swine (German Landrace, 104.4 ± 13.0 kg) before left ventricular assist device implantation. Hemodynamic data was recorded continuously, before and during EE. The herein described echocardiographic measurements were acquired according to a standardized protocol, encompassing apical, left ventricular short axis and long axis as well as epiaortic windows. In total, 50 echocardiographic parameters and 10 hemodynamic parameters were assessed.

**Results:**

Epicardial echocardiography was successfully performed in all animals, with a median screening time of 14 min (interquartile range 11–18 min). Referring to left ventricular function, ejection fraction was 51.6 ± 5.9% and 51.2 ± 6.2% using the Teichholz and Simpson methods, respectively. Calculated ventricular mass was 301.1 ± 64.0 g, as the left ventricular end-systolic and end-diastolic diameters were 35.3 ± 2.5 mm and 48.2 ± 3.5 mm, respectively. The mean heart rate was 103 ± 28 bpm, mean arterial pressure was 101 ± 20 mmHg and mean flow at the common carotid artery was 627 ± 203 mL/min.

**Conclusion:**

Epicardial echocardiography allows comprehensive assessment of most common echocardiographic parameters. Compared to humans, there are important differences in swine with respect to ventricular mass, size and wall thickness, especially in the right heart. Most hemodynamic parameters were comparable between swine and humans. This data supports study planning, animal and device selection, reinforcing the three R principles in animal research.

**Graphical Abstract:**

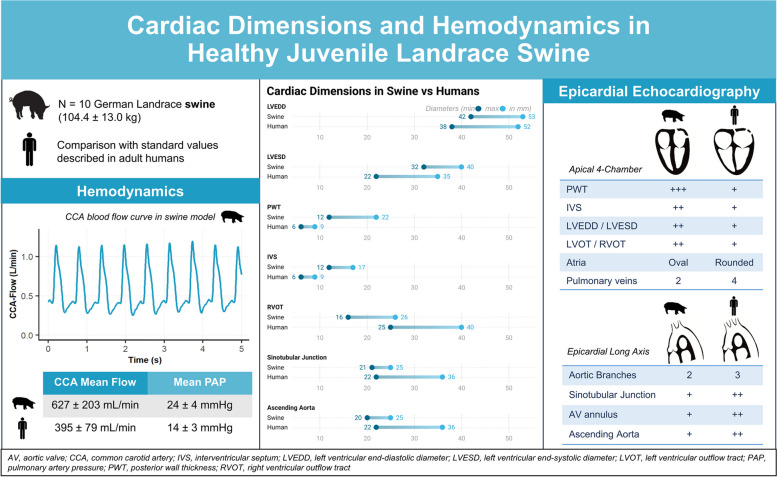

## Introduction

Swine are the preferred animal model for cardiovascular research, as they most closely represent human cardiac size, coronary anatomy and electrophysiology [1–3]. Those similarities, amongst limited differences, characterize this animal model as very representative of the conditions expected to be found in different scenarios of human cardiovascular surgery. Swine are used for hands-on training of young surgeons, as a disease-like model for high prevalent cardiovascular pathologies as well as for the development and testing of new medical devices and techniques. Given that, qualitative and quantitative data about the procedure and surgical outcomes is of vital relevance to the research scenario. In view of that, complementary to the ideal animal model is the choice of the most appropriate imaging method.

Epicardial echocardiography (EE) is the modality of ultrasonography performed with the transducer in direct contact with the cardiac surface. Historically, EE was first described in 1972, applied during mitral valve repairs [4], and the technique has been continuously evolved since then, providing lately comprehensive assessments with color flow and spectral Doppler. Epicardial echocardiography has a broad usage particularly in pediatric cardiac surgery, as it displays the anterior mediastinal structures in an enhanced manner [5, 6], and as TEE is not always safe in small children or patients with abnormalities of the great vessel [7]. Even though EE is a practical assessment tool, no standard values have been established for swine so far [8].

## Aims and objectives

The validation of EE as assessment tool for large animal models can support the development and testing of multiple medical devices, e.g., left ventricular assist devices, stents, grafts, and transcatheter minimal invasive valve repairs. Although TEE is the gold standard for intraoperative imaging in cardiac surgery, it is not always available, demands dedicated equipment and technique and, furthermore, requires advanced training and expertise. To the best of our knowledge, there is so far no study describing EE as a quantitative assessment tool, nor the report of a geometric baseline using this technique, particularly in swine.

The aim of this study is to describe our experience performing epicardial echocardiography in German Landrace swine, and the therefrom-acquired geometric and functional data. Furthermore, we aim to compare this data with the existent standard values from other techniques with traditional transthoracic and transesophageal echocardiography. With that, we wish to better understand the feasibility and accuracy of EE as a more practical quantitative assessment tool.

## Materials and methods

This study is part of a series of acute animal tests to validate a novel accessory for minimal-invasive implantation of a left ventricular assist device (LVAD). All experiments were approved by the local ethics committee (Freiburg, Germany, approval number 35–9185.81/G-22/006). All animals received human care in compliance with the *Guide for the Care and Use of Laboratory Animals* prepared by the Institute of Laboratory Animal Resources published by the National Institutes of Health. The first three animals were employed for developing and refinement of the methodology. Thereafter, ten healthy female swine (German Landrace, 104.4 ± 13.0 kg) were included in this study.

Before the procedures, all animals were kept under controlled environmental conditions. All animals received premedication and, after relaxation, were intubated and then transferred to the operating room. Premedication was induced with ketamine (20 mg/kg IM) and midazolam (0.5 mg/kg IM). After sedation, anesthesia was induced with propofol (2‒4 mg/kg IV) and vecuronium (0.2 mg/kg IV), and thereafter maintained with propofol (10–15 mg/kg/h IV), fentanyl (5–10 µg/kg IV) and vecuronium (0.2–0.4 mg/kg IV). All animals were placed in dorsal recumbency and continuously hemodynamic monitored for heart rate (HR), respiratory rate, peripheral oximetry (SpO_2_), arterial blood pressure (systolic, SBP; diastolic, DBP; mean, MAP), central venous pressure (CVP), pulmonary arterial pressure (systolic, sPAP; diastolic, dPAP; mean, mPAP) and electrocardiography. Blood flow through the left common carotid artery (CCA) was periodically assessed applying a transit-time flow meter (Medistim Deutschland GmbH, Deisenhofen, Germany).

**Fig. 1:**
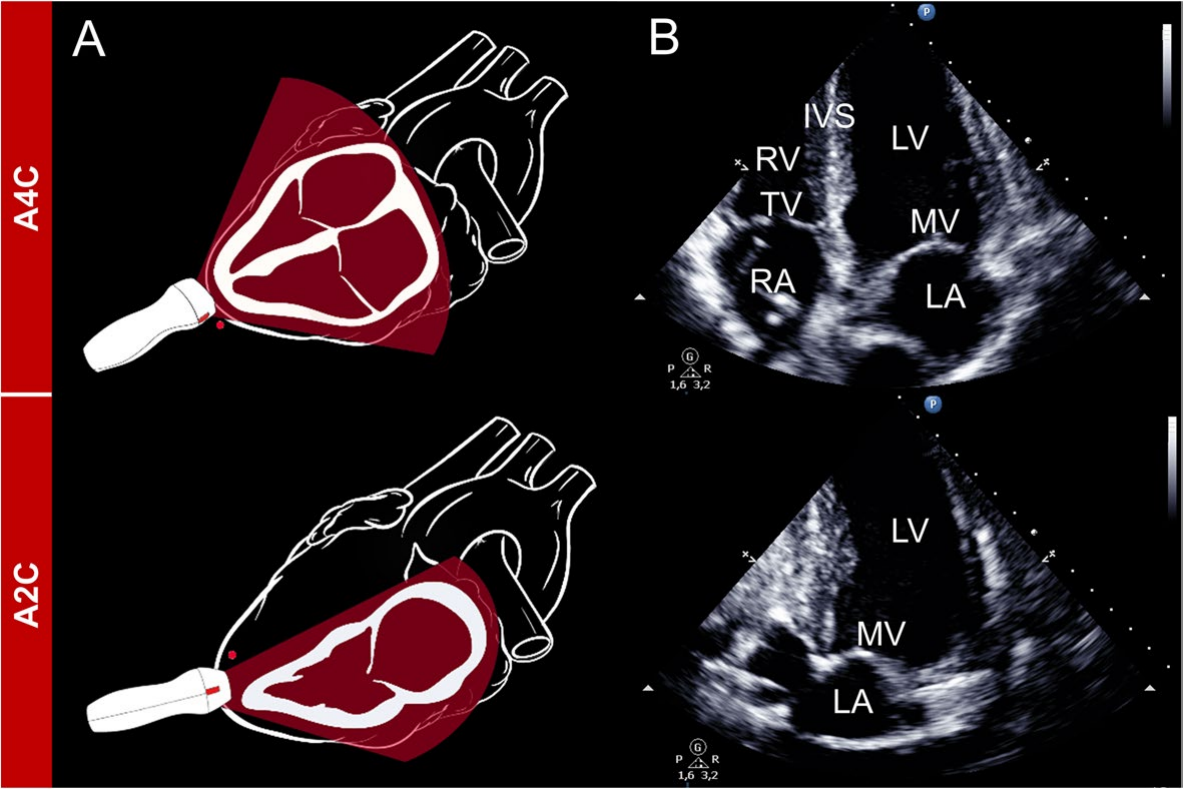
Apical 4- and 2-Chamber views. **A** refers to the schematic representation of the probe placement and structures to be displayed **B** represents the echocardiographic view. A2C, apical 2-Chamber; A4C, apical 4-Chamber; IVS, interventricular septum; LA, left atrium; LV, left ventricle; MV, mitral valve; RA, right atrium; RV, right ventricle; TV, tricuspid valve

**Fig. 2:**
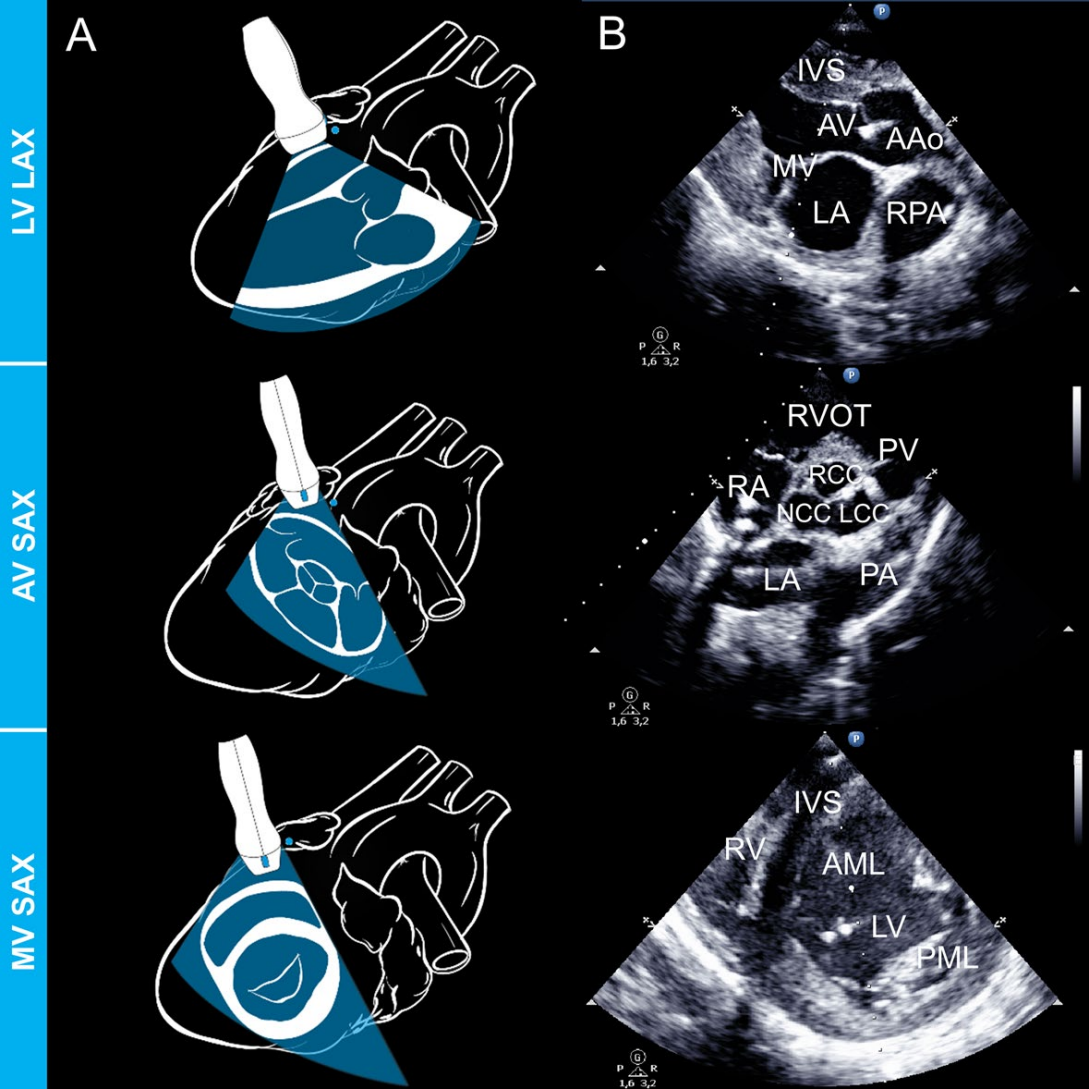
Ventricular LAX and SAX views. **A** refers to the schematic representation of the probe placement and structures to be displayed **B** represents the echocardiographic view. AAo, ascending aorta; AML, anterior mitral leaflet; AV, aortic valve; AV SAX, aortic alve short axis; IVS, interventricular septum; LA, left atrium; LCC, left coronary cusp; LV, left ventricle; LV LAX, left ventricular long axis; MV, mitral valve; MV SAX, mitral valve short axis; NCC, non-coronary cusp; PA, main pulmonary artery; PML, posterior mitral leaflet; PV, pulmonary valve; RA, right atrium; RCC, right coronary cusp; RPA, right pulmonary artery; RV, right ventricle; RVOT, right ventricular outflow tract

**Fig. 3:**
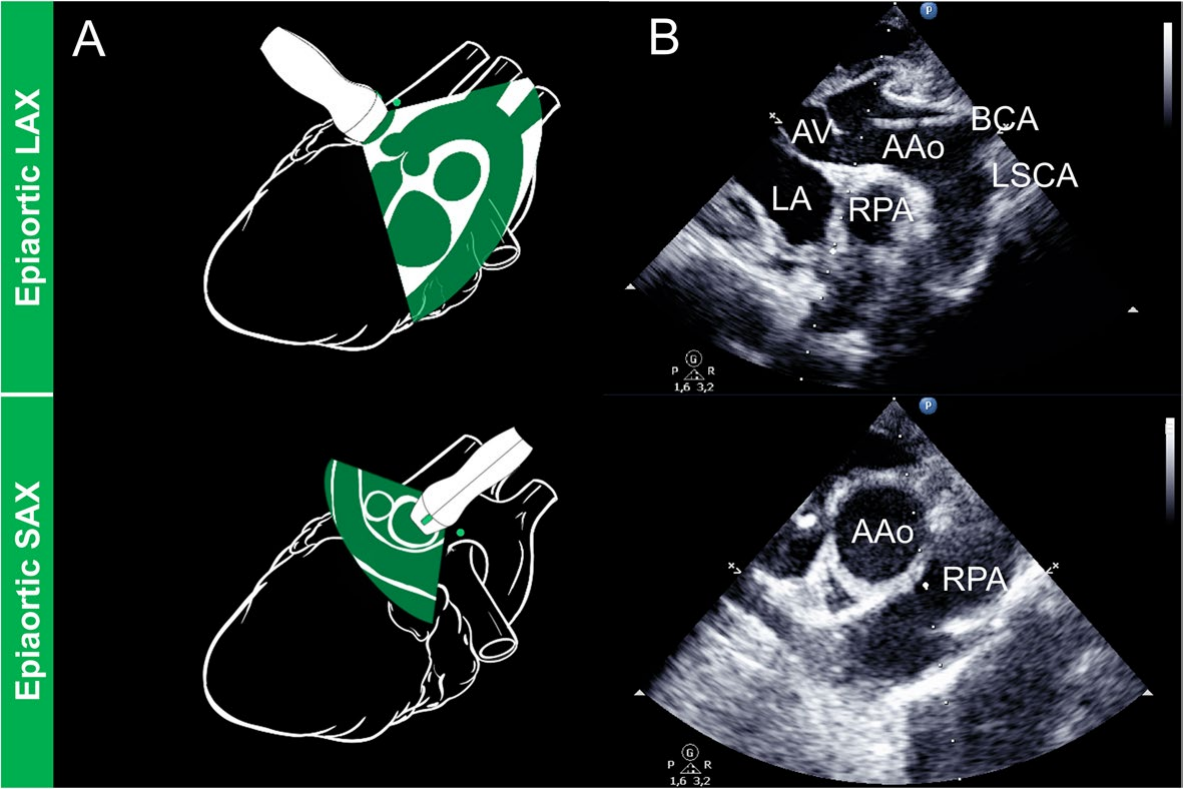
Epiaortic LAX and SAX views. **A** refers to the schematic representation of the probe placement and structures to be displayed **B** represents the echocardiographic view. AAo, ascending aorta; AV, aortic valve; BCA, brachiocephalic artery; LA, left atrium; LSCA, left subclavian artery; RPA, right pulmonary artery

Epicardial echocardiography was performed after sternotomy, before LVAD implantation. A broadband sector array transducer (S4-2, Philips CX50 Ultrasound Point of Care, Philips Healthcare, Hamburg, Germany) isolated by a sterile cover sheath was used to perform the screenings. The examinations followed an internal protocol for comprehensive epicardial echocardiography, encompassing apical 2- and 4-Chamber views (A2C and A4C, respectively) (Fig. 1), left ventricular long axis (LAX) and short axis (SAX) in different heights (Fig. 2), as well as epiaortic LAX and SAX views (Fig. 3).

All captions were initially assessed with two-dimensional (2D) ultrasound, in addition of color and spectral Doppler for valvular study, when applied. The diameters for the right ventricle (RV) and right atrium (RA), as well as the transvalvular hemodynamic parameters for the aortic and mitral valves (AV and MV, respectively) were assessed in the A4C view, as the ones for the left ventricle (LV), left atrium (LA) and ascending aorta (AAo) were measured in the LAX. The calculation of the sinotubular junction (STJ) height was performed at the center of cusp coaptation. The planimetry of the aortic valve (AV), the diameters of the mitral valve (MV), pulmonary artery (PA) and right ventricular outflow tract (RVOT) were assessed in the respective SAX height. The distances between each AV cusp and the contralateral commissure were analogously calculated at end-systole as previously described in magnetic resonance [9]. ejection fraction (EF) was calculated using the biplane Simpson method in A4C and A2C views, and comparatively applying the Teichholz method in the LV LAX.

Cardiac output (CO) and stroke volume were similarly obtained from the Simpson’s method. Diastolic dysfunction was evaluated according to the transmitral E/A ratio. The cardiac mass was defined according to the Devereux formula used as standard in humans as follows [10]:

$$LV\;mass=0.8 \times \left\{1.04 \times \left[{(LVEDD+PWT+IVS)}^{3}-{(LVEDD)}^{3}\right]\right\}+0.6 g$$

IVS, interventricular septum thickness; LV, left ventricle; LVEDD, left ventricular end-diastolic diameter; PWT, posterior wall thickness

All geometric parameters were measured post-operatively in three to five cardiac cycles. The examinations and measurements were performed uniformly in all animals. Data collection and statistical analysis were performed in a spreadsheet (Microsoft Excel, v. 2016), and further compared with the respective standard values established for humans. The hemodynamic mean values were calculated individually for each animal. The described means and standard deviations are referred to the individual means, in order to ensure sample uniformity. Quantitative data is presented as numbers and percentages. Values are shown as mean ± standard deviation (SD) and range, as well as median and interquartile range when applied.

## Results

### Vitals and hemodynamics

Baseline vital data of the subjects, from beginning of monitoring until sternotomy, is presented in Table 1.

**Table 1:** Baseline vital data in swine

	Landrace Swine *N* = 10	
Parameter	*Mean*	± *SD*
Heart Rate (bpm)	103	± 28
SBP (mmHg)	125	± 15
DBP (mmHg)	87	± 22
MAP (mmHg)	101	± 20
CVP (mmHg)	8	± 4
SpO_2_ (%)	99	± 1
sPAP (mmHg)	31	± 5
dPAP (mmHg)	17	± 5
mPAP (mmHg)	24	± 4
Mean CCA Flow (mL/min)	627	± 203

*CCA* Common carotid artery, *CVP* Central venous pressure, *DBP* Diastolic blood pressure, *dPAP* Diastolic pulmonary artery pressure, *MAP* Mean arterial pressure, *mPAP* Mean pulmonary artery pressure, *SBP* Systolic blood pressure, *sPAP* Systolic pulmonary artery pressure, *SpO2* Peripheral oxygen saturation

**Fig. 4:**
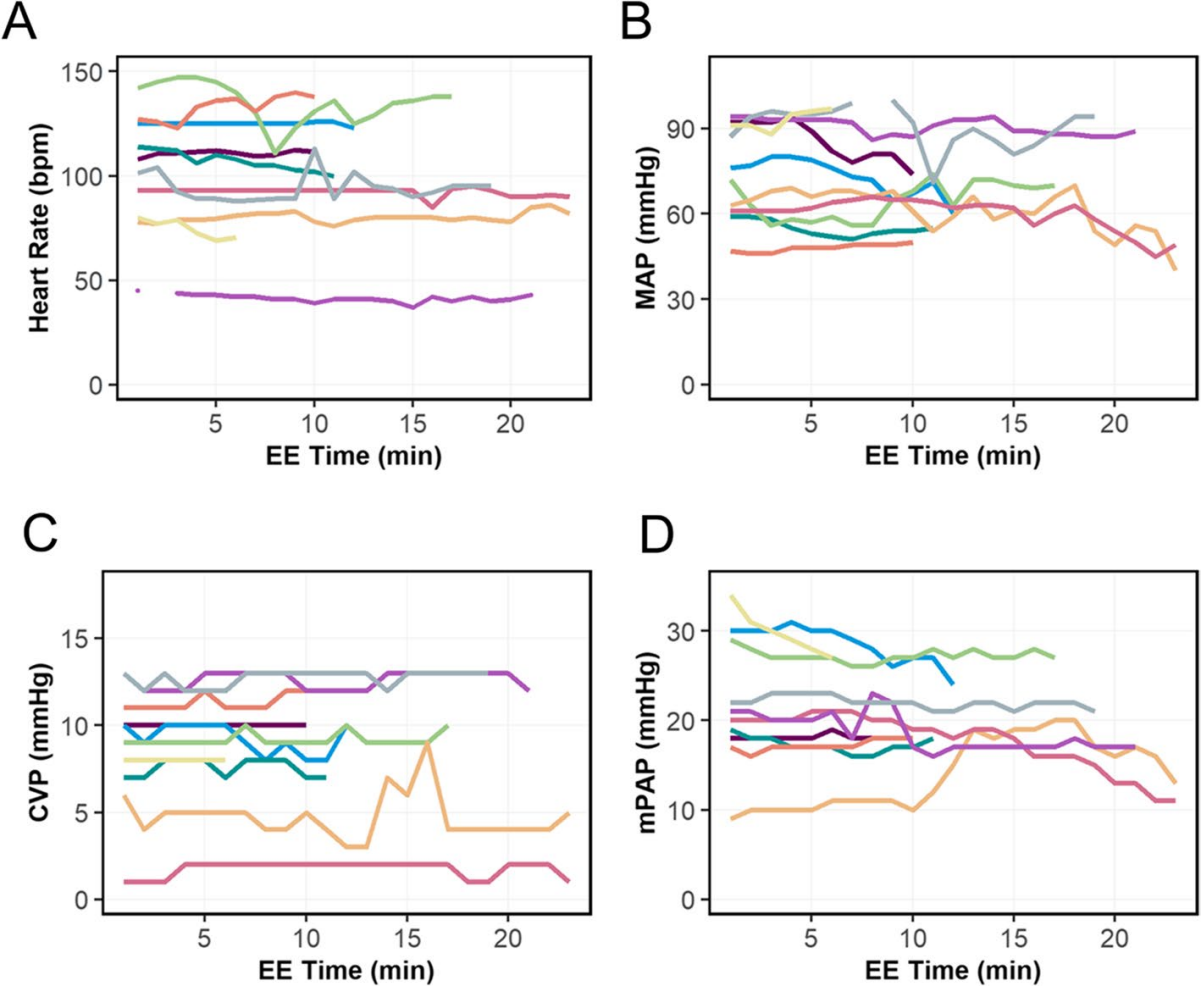
Vital parameters in swine during epicardial echocardiography. EE, epicardial echocardiography; CVP, central venous pressure; MAP, mean arterial pressure; mPAP, mean pulmonary artery pressure. The subjects marked in orange and red displayed a hypotensive tendency after minute 10 and 15 of the screenings, respectively. Sampling rate was achieved at 1:1 value per minute. **A**, heart rate **B**, mean arterial pressure **C**, central venous pressure **D**, mean pulmonary artery pressure

Epicardial echocardiography was successfully performed in all 10 animals, and the screening time varied between 6 and 23 min (median 14 min, interquartile range 11 – 18 min). The screenings did not induce significant changes on HR nor SpO_2_, which remained stable during the examinations. During EE, MAP was 72 ± 17 mmHg, and mean HR was 99 ± 29 beats per minute. Two animals displayed hypotensive tendency during the examinations, with MAP values ranging initially from 63 and 61 mmHg to 40 and 49 mmHg, respectively. One of them responded with higher values for mPAP and CVP. Figure 4 displays the values for HR, MAP, CVP and mPAP during EE examinations, respectively. Each line represents one subject. ^2^

### Left ventricle

Values for left ventricular dimensions and functions are summarized in Table 2.

**Table 2:** Left ventricular dimensions and function in swine vs. human reference

	Landrace Swine *N* = 10			Human Reference
Parameter	*Mean*	± *SD*	*Range*	*Range*
LVEDD (mm)	48.2	± 3.5	42.2–53.3	37.8–52.2^a^
LVESD (mm)	35.3	± 2.5	32.0–40.2	21.6–34.8^a^
LVEDV (mL)	85.5	± 10.9	69.0–107.0	46–106^a^
LVESV (mL)	44.7	± 10.1	30.6–67.5	14–42^a^
PWT (mm)	15.3	± 2.7	12.1–22.1	6–9^a^
IVS (mm)	14.0	± 1.5	11.7–16.7	6–9^a^
LVOT (mm)	21.5	± 1.5	18.7–23.4	17–25^b^
LV mass (g)	301.1	± 64.0	230.2–470.0	67–162^a^
EF Teichholz (%)	51.6	± 5.9	44.1–66.3	> 55^c^
EF Simpson (%)	51.2	± 6.2	66.5–63.5	54–74^a^
Stroke volume (mL)	40.8	± 7.7	29.4–53.2	50–100^c^
Cardiac output (L/min)	5.0	± 1.3	3.4–7.4	5.0–6.0^d^

*EF* Ejection fraction, *IVS* Interventricular septum thickness, *LVEDD* Left ventricular end-diastolic diameter, *LVESD* Left ventricular end-systolic diameter, *LVEDV* Left ventricular end-diastolic volume, *LVESV* Left ventricular end-systolic volume, *LVOT* Left ventricular outflow tract, *PWT* Posterior wall thickness

^a^Lang et al*.* [10]. Values are presented for females

^b^Kou et al*.* [11]

^c^Sidebotham *et. al*. [12]

^d^King and Lowery. [13]

### Right ventricle

Values for right ventricular dimensions and function are summarized in Table 3.

**Table 3:** Right ventricular dimensions and function in swine vs. human reference

	Landrace Swine *N* = 10			Human Reference
Parameter	*Mean*	± *SD*	*Range*	*Range*
RV systolic wall thickness (mm)	9.5	± 0.8	8.2–11.0	
RV diastolic wall thickness (mm)	6.7	± 0.7	5.4–7.6	1–5^a^
RV basal diameter (mm)	27.5	± 2.7	23.2–32.3	25–41^a^
RV mid diameter (mm)	15.0	± 2.0	11.6–18.0	19–35^a^
RV longitudinal diameter (mm)	65.5	± 4.8	59.4–75.4	59–83^a^
RVEDA (cm^2^)	9.6	± 2.0	5.7–13.1	6–13^b^
RVESA (cm^2^)	4.9	± 1.0	3.4–6.7	3–11^a^
FAC (%)	48.3	± 6.1	38.5–56.0	> 35^a^
TAPSE (mm)	18.7	± 4.6	13.7–27.2	> 17^a^
RVOT (mm)	21.0	± 2.8	16.4–25.5	25–40^b^
PA (mm)	21.2	± 2.0	18.3–23.6	11–31^e^

*FAC* Fractional area shortening, *PA* Pulmonary artery, *RV* Right ventricle, *RVEDA* Right ventricular end-diastolic area, *RVESA* Right ventricular end-systolic area, *RVOT* Right ventricular outflow tract, *TAPSE* Tricuspid annular plane systolic excursion

^a^Lang et al*.* [10]. Values are presented for females

^b^Kou et al*.* [11]

^e^Sheikhzadeh et al*.* [14]

### Left and right atria

Values for the left and right atria are summarized in Table 4.

**Table 4:** Atrial dimensions in swine vs. human reference

	Landrace Swine *N* = 10			Human Reference
Parameter	*Mean*	± *SD*	*Range*	*Range*
LA minor axis diameter (mm)	34.2	± 3.6	30.1–42.2	27–38^a^
LA major axis diameter (mm)	39.4	± 6.6	26.2–49.9	
LA area (cm^2^)	12.5	± 4.0	8.1–20.2	≤ 20^a^
RA minor axis diameter (mm)	29.6	± 3.7	24.9–34.7	< 18^f^
RA major axis diameter (mm)	39.4	± 4.6	30.8–47.6	
RA area (cm^2^)	10.0	± 2.3	7.1–13.9	10–20^b^

*LA* Left atrium, *RA* Right atrium

^a^Lang et al*.* [10]. Values are presented for females

^b^Kou et al*.* [11]

^f^Rudski et al*.* [15]

### Aortic and mitral valves

Regarding valvular function, mild aortic and mitral regurgitation were found in one and four animals at baseline, respectively. The mean values for the E and A waves were 82.7 ± 18.6 and 57.2 ± 13.4 cm/s, respectively. There were no signs of severe regurgitation in any evaluated valve in all animals. Further values for AV and MV are summarized in Table 5.

**Table 5:** Aortic and Mitral valve diameters and function in swine vs. human reference

	Landrace Swine *N* = 10			Human Reference
Parameter	*Mean*	± *SD*	*Range*	*Range*
***Aortic Valve***				
Annulus diameter (mm)	24.6	± 1.9	21.4–27.6	20–31^c^
Opening area (cm^2^)	5.2	± 0.8	4.2–6.7	2.3–4.1^g^
Peak velocity (m/s)	1.4	± 0.3	1.0–1.9	≤ 1.0^h^
VTI (cm)	26.7	± 4.3	20.3–33.7	17–34^i^
LCC-Commissure (mm)	29.7	± 2.5	24.9–33.3	
NCC-Commissure (mm)	30.5	± 2.5	27.1–35.1	
RCC-Commissure (mm)	27.9	± 2.4	23.7–31.1	
***Mitral Valve***				
E/A ratio	1.5	± 0.3	0.7–2.2	≥ 0.8^j^
Peak velocity (m/s)	1.3	± 0.4	0.5–1.9	0.6–0.8^j^
Deceleration time (ms)	145	± 31	102–190	150–240^j^
Anteroposterior diameter (mm)	25.8	± 4.9	19.9–36.1	25–38^k^
Intercommissural diameter (mm)	45.2	± 3.0	39.9–50.1	28–42^k^

*AV* Aortic valve, *LCC* Left coronary cusp, *NCC* Non-coronary cusp, *RCC* Right coronary cusp, *VTI* Velocity time integral

^c^Evangelista et al*.* [16]

^g^Pollick et al*.* [17]

^h^Chaothawee et al*.* [18]

^i^Cotella et al*.* [19]. Values are presented for females

^j^Nagueh et al*.* [20]

^k^Dwivedi et al*.* [21]. Values are presented for females

### Ascending aorta

Values for the ascending aorta are summarized on Table 6.

**Table 6:** Ascending aorta diameters in swine vs. human reference

	Landrace Swine *N* = 10			Human Reference
Parameter	*Mean*	± *SD*	*Range*	*Range*
Sinus of Valsalva (mm)	29.5	± 2.1	26.1–33.0	29–45^c^
STJ diameter (mm)	22.5	± 1.3	20.9–24.6	22–36^c^
STJ height (mm)	20.4	± 1.9	18.1–24.8	18–23^l^
STJ to BCA (mm)	37.2	± 5.6	30.5–45.8	
AAo diameter (mm)	23.1	± 1.4	20.0–25.1	22–36^c^
Aortic arch diameter (mm)	23.6	± 2.7	20.0–30.0	22–36^c^
RCA height	17.9	± 1.5	15.0–19.9	
Root perimeter (mm)	103.4	± 8.9	89.6–116.0	69–82^l^
Root area (cm^2^)	8.0	± 1.3	5.6–10.0	3.8–4.8^l^

*AAo* Ascending aorta, *BCA* Brachiocephalic artery, *RCA* Right coronary artery, *STJ* Sinotubular junction

^c^Evangelista et al*.* [16]

^l^Li et al*.* [22]. Values acquired by computer tomography

## Discussion

In this article, we described the measurements obtained from intraoperative EE in juvenile Landrace swine. This is of relevance as swine are the preferred large animal model for in vivo studies in cardiovascular research [1–3]. Swine share multiple similarities regarding anatomy and physiology with the human heart, which is of utmost relevance for animal testing, hands-on surgical training and development of medical devices (e.g., LVADs, transcatheter valve implantation, etc.). Such similarities, as well as the particularities of the swine heart, exemplary the LA receiving only two pulmonary veins and solely two branches arising from the aortic arch [23], can be well displayed through EE withing minutes of screening, requiring neither high-end equipment nor advanced experience. There is limited data concerning echocardiography in swine and, so far, no reports using epicardial and epiaortic screening. This raises concerns as the establishment of a geometric and functional baseline in swine could substantially reduce the number of required subjects in animal tests, contributing to reinforce the bioethical three R principles in research with animal models [24].

The vital data resembles the physiologic standard values in humans, especially regarding blood pressure, CVP and and SpO_2_. The transit-time CCA flow measurement, assessed by duplex sonography, was of 627 ± 203 mL/min, values notably higher as the ones so far described in humans [25, 26]. We hypothesize that this could be related to the stronger head and neck musculature development compared to humans, as well as to the quadruped posture in swine, favorizing the blood propelling as by enduring smaller gravitational force. Values for mean PAP were likewise higher than the standard values described in humans (14.0 ± 3.3 mmHg) [27, 28].

The swine heart displays a typically bulkier myocardium, with pronounced ventricular walls. This is remarkably demonstrated by the greater values found for PWT and IVS, as well as the higher calculated LV mass. Comparatively, the values encountered about EF were smaller as the standard human values. The thicker aspect of the myocardium is also demonstrated by broader RV walls. This can be attributed to the profuse myocardial tissue constituting the ventricular walls. Analogously, the diameters of the RVOT and PA were smaller as the human standards. We hypothesize that this may be associated with the aforementioned bulkier myocardium, also implying greater contractility in the right heart, or to higher pressure gradients in the pulmonary circulation in swine. Both atria portray an oval shape as previously described in swine [8, 29], with substantial greater lengths in the long axis in comparison with the short axis of each chamber.

Regarding ventricular function, there was no noteworthy difference between EF assessment via modified Simpson or Teichholz methods. This may be of importance when considering the sensible and variable geometry of human hearts, setting the Simpson method as gold standard [10], which requires particular technical refinement. Our data shows that both techniques are applicable to swine, what may be of advantage when the Simpson method is not available or not viable. This representative assessment of the Teichholz technique in swine may be associated with their aforementioned bulkier myocardium, sculpting the heart with a bullet-shaped form, which is the essential assumption of the Teichholz technique [30].

The transversal diameters encountered for the aortic root (Sinus of Valsalva and STJ) were encompassed in the lower range of the standard values in humans. However, the diameters of the proximal ascending aorta and aortic arch were smaller as the standard human values. In addition to the classic echocardiographic parameters, we assessed the distance between the STJ and the first aortic branch, namely brachiocephalic artery in swine. To the best of our knowledge, such data has not been reported using EE until this point. Likewise, we reported values for the height of the RCA’s ostium. We found one study reporting this parameter with echocardiography in horses [31]. The contemporary data may be of special importance to transcatheter procedures, arterial accesses to the heart using the common carotid artery, as well as procedures with replacement of the aortic root, which could interfere with the coronary circulation.

Regarding transvalvular parameters, the mitral valve E/A ratio resembles the physiologic values found in humans, as the peak velocities for both AV and MV are noticeably higher [18, 20]. This may represent a hyperdynamic status from swine’ cardiovascular system, enclosing greater contraction forces propelling the blood through the cardiac chambers.

With respect to further echocardiographic reports in animal models, Sündermann et al*.* described a geometric and functional baseline by performing a similar study using TEE in 20 domestic swine (56–106 kg) [32]. With exception of the lower ranges attributed to smaller subjects, we found multiple comparable results using EE. Analogously, Huenges et al*.* performed TEE in 45 German Landrace swine (46–57.5 kg) with focused assessment on global heart function, valvular function and detection of possible regurgitation [8]. Their screening was particular for including advanced hemodynamic parameters, such as transvalvular patterns, velocities, pressure gradients and velocity time integrals over the LVOT and both AV and MV. We found comparable results regarding EF using different methods, as well as akin E/A ratio and LVOT diameters. Unlike the two aforementioned reports, we found higher values for the maximal and average heart rate. This may be attributed to the transmural Purkinje fiber distribution in the porcine heart, associated with the higher susceptibility to tachyarrhythmias presented by this species [1, 3].

Epicardial echocardiography is often described as a qualitative assessment tool but, to the best of our knowledge, until today there is no data referring to quantitative parameters and measurements performed using such method. The epicardial technique regained certain highlight after the Covid-19 pandemics, by providing intraoperative imaging with little to no aerosolization, easy disinfection and no direct contact with patient’s body fluids [33]. Furthermore, as much as TEE is the gold standard intraoperative imaging method, in particular situations such as cardiac interventions in congenital pathologies, EE is considered a complementary tool to TEE, rather than two distinct methods [5, 34]. Additionally, EE provides superior imaging of anterior vascular structures, allowing direct vessel visualization and adequate Doppler alignment, with low complication rates [5, 35].

Although TEE is the standard intraoperative imaging modality and EE has logistic and technical advantages, one technique does not preclude another. There are reports where both techniques are combined, providing suitable results [5, 6, 34]. Epicardial echocardiography showed to be applicable in the swine model and may support TEE whenever the probe placement is not successful or contraindicated, as well as enrich this modality with comparable data.

## Conclusion

In this study, we report the a comprehensive dataset obtained from intraoperative EE in swine. In total, 50 echocardiographic parameters are described, from which most were comparable to humans. We believe that the establishment of a detailed geometric and functional baseline in swine using EE is of great relevance and practical execution, and may greatly support the current standard of cardiovascular research using swine models, reinforcing the bioethical principles of the three R principles – Reducment, Replacement and Refinement.

## Data Availability

The data that support the findings of this study are available from the corresponding author, [WB], upon reasonable request.

## Abbreviations

2D: Two-dimensional
AAo: Ascending aorta
AV: Aortic valve
CCA: Common carotid artery
CVP: Central venous pressure
DBP: Diastolic blood pressure
dPAP: Diastolic pulmonary artery pressure
EE: Epicardial echocardiography
EF: Ejection fraction
FAC: Fractional area changing
HR: Heart rate
IVS: Interventricular septum
LA: Left atrium
LAX: Long axis
LCC: Left coronary cusp
LV: Left ventricle
LVAD: Left ventricular assist device
LVEDD: Left ventricular end-diastolic diameter
LVESD: Left ventricular end-systolic diameter
LVOT: Left ventricular outflow tract
MAP: Mean arterial pressure
mPAP: Mean pulmonary artery pressure
MV: Mitral valve
NCC: Non-coronary cusp
PA: Pulmonary artery
PWT: Posterior wall thickness
RA: Right atrium
RCA: Right coronary artery
RCC: Right coronary cusp
RV: Right ventricle
RVOT: Right ventricular outflow tract
SAX: Short axis
sPAP: Systolic pulmonary artery pressure
SPB: Systolic arterial pressure
SpO_2_: Peripheral oxygen saturation
STJ: Sinotubular junction
SD: Standard deviation
TEE: Transesophageal echocardiography
TTE: Transthoracic echocardiography
VTI: Velocity time integral
